# Long-term clinical impact of permanent pacemaker implantation in patients undergoing transcatheter aortic valve implantation: a systematic review and meta-analysis

**DOI:** 10.1093/europace/euac008

**Published:** 2022-02-09

**Authors:** Andrea Zito, Giuseppe Princi, Marco Lombardi, Domenico D’Amario, Rocco Vergallo, Cristina Aurigemma, Enrico Romagnoli, Gemma Pelargonio, Piergiorgio Bruno, Carlo Trani, Francesco Burzotta, Filippo Crea

**Affiliations:** Department of Cardiovascular and Thoracic Sciences, Università Cattolica del Sacro Cuore, L.go A. Gemelli 1, 00168 Rome, Italy; Department of Cardiovascular and Thoracic Sciences, Università Cattolica del Sacro Cuore, L.go A. Gemelli 1, 00168 Rome, Italy; Department of Cardiovascular and Thoracic Sciences, Università Cattolica del Sacro Cuore, L.go A. Gemelli 1, 00168 Rome, Italy; Department of Cardiovascular and Thoracic Sciences, Università Cattolica del Sacro Cuore, L.go A. Gemelli 1, 00168 Rome, Italy; Department of Cardiovascular Medicine, Fondazione Policlinico Universitario A. Gemelli IRCCS, Rome, Italy; Department of Cardiovascular and Thoracic Sciences, Università Cattolica del Sacro Cuore, L.go A. Gemelli 1, 00168 Rome, Italy; Department of Cardiovascular Medicine, Fondazione Policlinico Universitario A. Gemelli IRCCS, Rome, Italy; Department of Cardiovascular and Thoracic Sciences, Università Cattolica del Sacro Cuore, L.go A. Gemelli 1, 00168 Rome, Italy; Department of Cardiovascular Medicine, Fondazione Policlinico Universitario A. Gemelli IRCCS, Rome, Italy; Department of Cardiovascular and Thoracic Sciences, Università Cattolica del Sacro Cuore, L.go A. Gemelli 1, 00168 Rome, Italy; Department of Cardiovascular Medicine, Fondazione Policlinico Universitario A. Gemelli IRCCS, Rome, Italy; Department of Cardiovascular and Thoracic Sciences, Università Cattolica del Sacro Cuore, L.go A. Gemelli 1, 00168 Rome, Italy; Department of Cardiovascular Medicine, Fondazione Policlinico Universitario A. Gemelli IRCCS, Rome, Italy; Department of Cardiovascular and Thoracic Sciences, Università Cattolica del Sacro Cuore, L.go A. Gemelli 1, 00168 Rome, Italy; Department of Cardiovascular Medicine, Fondazione Policlinico Universitario A. Gemelli IRCCS, Rome, Italy; Department of Cardiovascular and Thoracic Sciences, Università Cattolica del Sacro Cuore, L.go A. Gemelli 1, 00168 Rome, Italy; Department of Cardiovascular Medicine, Fondazione Policlinico Universitario A. Gemelli IRCCS, Rome, Italy; Department of Cardiovascular and Thoracic Sciences, Università Cattolica del Sacro Cuore, L.go A. Gemelli 1, 00168 Rome, Italy; Department of Cardiovascular Medicine, Fondazione Policlinico Universitario A. Gemelli IRCCS, Rome, Italy; Department of Cardiovascular and Thoracic Sciences, Università Cattolica del Sacro Cuore, L.go A. Gemelli 1, 00168 Rome, Italy; Department of Cardiovascular Medicine, Fondazione Policlinico Universitario A. Gemelli IRCCS, Rome, Italy

**Keywords:** Permanent pacemaker implantation, Transcatheter aortic valve implantation, Transcatheter aortic valve replacement, Clinical outcome, Personalized medicine, Meta-analysis

## Abstract

**Aims:**

The aims of this study is to assess by an updated meta-analysis the clinical outcomes related to permanent pacemaker implantation (PPI) after transcatheter aortic valve implantation (TAVI) at long-term (≥12 months) follow-up (LTF).

**Methods and results:**

A comprehensive literature research was performed on PubMed and EMBASE. The primary endpoint was all-cause death. Secondary endpoints were rehospitalization for heart failure, stroke, and myocardial infarction. A subgroup analysis was performed according to the Society of Thoracic Surgeon—Predicted Risk of Mortality (STS-PROM) score. This study is registered with PROSPERO (CRD42021243301). A total of 51 069 patients undergoing TAVI from 31 observational studies were included. The mean duration of follow-up was 22 months. At LTF, PPI post-TAVI was associated with a higher risk of all-cause death [risk ratio (RR) 1.18, 95% confidence interval (CI) 1.10–1.25; *P* < 0.001] and rehospitalization for heart failure (RR 1.32, 95% CI 1.13–1.52; *P* < 0.001). In contrast, the risks of stroke and myocardial infarction were not affected. Among the 20 studies that reported procedural risk, the association between PPI and all-cause death risk at LTF was statistically significant only in studies enrolling patients with high STS-PROM score (RR 1.25, 95% CI 1.12–1.40), although there was a similar tendency of the results in those at medium and low risk.

**Conclusion:**

Patients necessitating PPI after TAVI have a higher long-term risk of all-cause death and rehospitalization for heart failure as compared to those who do not receive PPI.

## Graphical Abstract

**Graphical Abstract:**
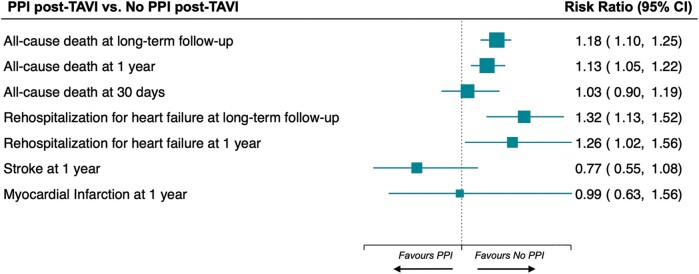

What’s new? Since the first clinical report in 2002, transcatheter aortic valve implantation (TAVI) has emerged as a worthy, less-invasive, and safe alternative for the therapeutic management of patients with severe aortic stenosis (AS).^1^ Over the years, TAVI gained the role of treatment of choice in inoperable patients and those at high or intermediate surgical risk.^2^ More recently, two randomized controlled clinical trials (RCTs) have supported the indication for TAVI even in patients at low surgical risk.^3,^^4^

- Transcatheter aortic valve implantation (TAVI), compared with surgery, led to an increased need for post-operative permanent pacemaker implantation (PPI).
- In this meta-analysis including 51 069 patients across 31 observational studies, PPI post-TAVI was associated with an increased long-term risk of all-cause death and rehospitalization for heart failure.
- These results help to characterize the prognosis of patients undergoing TAVI.

## Introduction

Among different complications that can occur after TAVI, the development of conduction abnormalities is extremely frequent.^5^ An injury to the atrioventricular conduction system during balloon valvuloplasty or prosthesis implantation and ischaemia of the conduction pathways can lead to advanced conduction disorders that often require permanent pacemaker implantation (PPI).^6^

The incidence of post-procedural PPI is higher after TAVI compared with surgical aortic valve replacement (SAVR); in particular, a recent RCT showed that PPI occurred in 33% of TAVI and 20% of SAVR patients at 5 years of follow-up.^7^

The need for PPI in patients undergoing TAVI is known to be influenced by both clinical and technical aspects.^8^ However, to date, the prognostic impact of PPI after TAVI is still debated; indeed, recent observational data have reported conflicting results.^9–13^ Furthermore, previous meta-analyses (with a small number of studies included, different adjudication methods of the outcome of interest, and short-term follow-up period) have yielded jarring results.^14–19^

On such bases, we performed the present systematic review and meta-analysis to evaluate the impact of PPI on long-term clinical outcomes of patients with AS undergoing TAVI.

## Methods

This meta-analysis was carried out in accordance with the Meta-analysis Of Observational Studies in Epidemiology (MOOSE) guidelines^20^ and was registered within the PROSPERO International Prospective Register of Systematic Reviews (CRD42021243301).

### Search strategy and selection criteria

A systematic and comprehensive literature research was performed on PubMed and EMBASE databases, from inception to October 2021, to identify studies that investigated the impact of PPI after TAVI on clinical outcomes. We used a combination of the following keywords and MeSH terms: TAVI, PPI, mortality. The full research strategy is listed in Supplementary material online, *Table S1*.

All records retrieved from the research were systematically screened in parallel and independently by two authors (A.Z. and G.P.), according to titles and abstracts; conflicts were resolved by collegial discussion.

The following inclusion criteria were used: (i) studies reporting adverse events related to PPI after TAVI in native valves and (ii) studies reporting long-term clinical outcomes (follow-up ≥ 12 months).

We excluded studies in which outcomes of interest were not clearly reported or were impossible to extract from the published results, studies that included patients with pacemaker before TAVI, conference abstracts, comments, editorials, case reports, systematic reviews, and meta-analysis.

When two or more studies were reported from the same cohort of subjects, the most recent publication or the one with the longest follow-up was included in the analysis.

### Data extraction and quality assessment

Data extraction from the studies included was performed independently by two coauthors (A.Z. and G.P.) using a standardized worksheet. If available, the following items were collected: first author’s name, year of publication, study design, region, number of centres where the study was carried out, sample size, incidence of PPI, timing of PPI, baseline demographic and clinical characteristics of the population, valve type implanted, Society of Thoracic Surgeons—Predicted Risk of Mortality (STS-PROM) score,^21^ and follow-up duration.

Particularly, the STS-PROM score is a validated risk prediction model based on ∼50 clinical pre-operative variables from the STS National Adult Cardiac Surgery Database such as age, race, cardiovascular risk factors, clinical presentation; it allows to calculate a patient’s risk of mortality for both the most commonly performed cardiac surgeries and TAVI.

Finally, the data necessary for the outcome analysis were also extracted; data at 1-year follow-up that were not directly available were retrieved from another meta-analysis that previously retrieved data from the corresponding author.^14^

Quality assessment of the studies was made independently by two coauthors (A.Z. and G.P.) using the standardized Newcastle–Ottawa Scale (NOS),^22^ producing a quality score (from 0 to 9) for each study included.

### Study endpoints

The primary endpoint of the study was all-cause death at long-term (≥ 12 months) follow-up (LTF). The risk of all-cause death was calculated at 30 days and 1 year to assess the possible impact of follow-up duration.

Secondary endpoints were rehospitalization for heart failure, stroke, and myocardial infarction at LTF and 1 year.

### Statistical analysis

Categorical dichotomous data were summarized across treatment arms, compared, and reported as crude risk ratio (RR) with the corresponding 95% confidence interval (CI). As primary analysis, we used DerSimonian and Laird random-effects model. As secondary analyses, we also reported effects estimates as crude odds ratio (OR) with the corresponding 95% CI and computed Mantel–Haenszel fixed-effects model. We evaluated heterogeneity of effects using the Cochran *Q* test statistic and Higgins and Thompson *I*^2^. According to prespecified cutoffs, low heterogeneity was defined as an *I*^2^ <25%, moderate heterogeneity as an *I*^2^ between 25% and 75%, and high heterogeneity as an *I*^2^ >75%. We visually inspected funnel plots for asymmetry and used Egger’s regression asymmetry test to assess the potential effect of publication bias. Furthermore, we performed a subgroup analysis stratifying studies according to the mortality risk of patients predicted by the STS-PROM score [high risk of mortality (≥8%), intermediate risk (4–8%), and low risk (<4%), as previously reported]^23^ to evaluate whether the impact of PPI after TAVI on all-cause death at LTF was influenced by this variable.

Sensitivity analyses were performed by comparing the results of the primary and secondary analyses. In order to investigate potential sources of heterogeneity for the LTF outcomes, we performed several univariable random-effects meta-regression analyses with the DerSimonian and Laird method according to age, sex category, atrial fibrillation, diabetes mellitus, coronary artery disease (CAD), left ventricular ejection fraction (LVEF), number of self-expanding and balloon-expanding valves implanted, NOS, and duration of follow-up. Secondary and subgroup analyses were not prespecified. Descriptive characteristics were presented as mean ± standard deviation or median (inter-quartile range) for continuous variables and as frequencies and percentages for categorical variables. Statistical analysis was performed using Stata 17 (StataCorp).

## Results

The initial search retrieved 2066 records (616 from PubMed and 1450 from EMBASE). 2003 records were excluded because of different study design or topic of interest after the evaluation of titles and abstracts. Then, other 63 records were subsequently excluded after full-text assessment. Finally, 31 records were selected (Supplementary material online, *Table S2*), with 51 069 patients undergoing TAVI included in the analysis. The mean follow-up duration was of 22 months (range 12–60 months).

Study and population characteristics are summarized in *Table 1* and Supplementary material online, *Table S3*.

**Table 1:** Main characteristics of the studies included in the systematic review

Author	Year	Type of study	Region	Centres	Inclusion period	No. of patients	No. of patients undergoing PPI (%)	Timing of PPI	Type of valve implanted (%)	Follow-up (months)
Alasti *et al*.^24^	2018	Obs, prospective	Australia	1	April 2012–October 2016	152	38 (25.0)	Within 30 days	MEV (100)	12
Aljabbary *et al.*^25^	2018	Obs, retrospective	Canada	10	April 2010–October 2015	1263	186 (14.7)	During hospitalization	NA	33
Ashraf *et al*.^12^	2020	Obs, retrospective	Arizona	1	January 2012–July 2018	243	22 (9.1)	Within 30 days	BEV (100)	36
Biner *et al*.^26^	2014	Obs, retrospective	Israel	1	NA	230	58 (25.4)	NA	SEV (87.4)	19.5
									BEV (12.6)	
Buellesfeld *et al*.^27^	2012	Obs, prospective	Switzerland, Germany	2	August 2007–March 2010	305	98 (32.1)	Within 30 days	SEV (89.5)	12
									BEV (10.5)	
Chamandi *et al*.^28^	2018	Obs, prospective	International	9	May 2007–February 2011	1629	322 (19.8)	Within 30 days	SEV (53.9)	52
									BEV (43.8)	
Costa *et al*.^10^	2019	Obs, prospective	Italy	1	June 2007–February 2018	1116	145 (13.0)	Within 30 days	SEV (72.5)	12
									BEV (27.2)	
D’Ancona *et al*.^29^	2011	Obs, prospective	Germany	1	April 2008–March 2011	322	20 (6.2)	Within 30 days	BEV (100)	12
De Carlo *et al*.^30^	2011	Obs, prospective	Italy	3	September 2007–July 2010	275	66 (24.0)	0–2 days	SEV (100)	12
Du *et al*.^11^	2019	Obs, retrospective	China	1	March 2013–October 2018	256	38 (14.8)	Within 30 days	SEV (100)	12
Engborg *et al*.^31^	2016	Obs, prospective	Denmark	1	March 2008–September 2012	128	41 (32.0)	Within 30 days	SEV (78.1)	46.2
									BEV (21.9)	
Fadahunsi *et al*.^32^	2016	Obs, retrospective	USA	220	November 2011–September 2014	9785	651 (6.7)	Within 30 days	SEV (11.2)	12
									BEV (88.8)	
Fujita *et al*.^33^	2019	Obs, prospective	Germany	Multicentre	2011–15	20 872	3459 (16.6)	During hospitalization	SEV (36.0)	12
									BEV (53.7)	
									DFM (1.5)	
Gensas *et al*.^34^	2014	Obs, retrospective	Brazil	18	January 2008–February 2012	353	89 (25.2)	Within 30 days	SEV (85.8)	60
									BEV (14.2)	
Giustino *et al*.^35^	2016	Obs, retrospective	Europe	4	November 2005–December 2011	947	145 (13.2)	Within 30 days	SEV (52.1)	60
									BEV (47.9)	
Gonska *et al*.^36^	2018	Obs, retrospective	Germany	1	February 2014–September 2016	612	168 (27.5)	NA	SEV (4.4)	12
									BEV (58.8)	
									MEV (36.8)	
Houthuizen *et al*.^37^	2012	Obs, prospective	Netherlands	8	November 2005–December 2010	797	118 (14.8)	Within 30 days	SEV (61.4)	15
									BEV (38.6)	
Jørgensen *et al*.^38^	2019	Obs, prospective	Denmark	1	2007–17	816	132 (16.2)	Within 30 days	SEV (82.6)	30
									BEV (9.4)	
									MEV (8.0)	
Kostopoulou *et al*.^39^	2015	Obs, prospective	Greece	1	January 2010–February 2012	45	10 (22.2)	Within 30 days	SEV (100)	24
López-Aguilera *et al.*^40^	2018	Obs, prospective	Spain	1	April 2008–December 2015	217	39 (15.0)	During hospitalization	SEV (100)	37
Meduri *et al*.^9^	2019	Obs, prospective	North America, Europe, Australia	55	September 2014–December 2015	704	245 (34.8)	Within 30 days	SEV (33.8)	12
									MEV (66.2)	
Mouillet *et al*.^41^	2015	Obs, prospective	International	29	January 2010–October 2011	883	252 (30.3)	Within 1 year	SEV (100)	12
Nadeem *et al*.^42^	2018	Obs, retrospective	Ohio	1	2011 – 2017	672	146 (21.7)	Within 1 year	SEV (55.5)	12
									BEV (44.2)	
Nazif *et al*.^43^	2015	Obs, retrospective	International	21	May 2007–September 2011	1973	173 (8.8)	Within 30 days	BEV (100)	12
Nijenhuis *et al.*^44^	2017	Obs, retrospective	Netherlands	1	June 2007–June 2015	155	37 (23.9)	Within 30 days	NA	18.6
Pereira *et al*.^45^	2013	Obs, retrospective	Portugal	1	August 2007–May 2011	58	19 (32.8)	During hospitalization	SEV (100)	12
Rogers *et al*.^46^	2018	Obs, prospective	USA	1	January 2013–December 2015	614	145 (23.6)	Within 30 days	SEV (22.0)	12
									BEV (78.0)	
Rück *et al*.^13^	2021	Obs, population-based cohort	Sweden	8	January 2008–December 2018	3420	481 (14.1)	Within 30 days	BEV (38.4)	32.4 (20.4 m for HF outcome)
Schymik *et al*.^47^	2015	Obs, retrospective	Germany	1	May 2008–April 2012	634	69 (10.8)	Within 24 h	SEV (19.2)	12
									BEV (80.8)	
Urena *et al*.^48^	2014	Obs, retrospective	International	8	January 2005–February 2013	1556	239 (15.4)	Within 30 days	SEV (44.9)	22
									BEV (55.1)	
Walther *et al.*^49^	2018	Obs, prospective	Europe, Australia	12	December 2011–September 2015	198	29 (14.7)	During hospitalization	SEV (100)	12

BEV, balloon-expanding valves; HF, heart failure; MEV, mechanically expandable valves; NA, not available; Obs, observational; PPI, permanent pacemaker implantation; SEV, self-expandable valves.

All studies were of observational nature, of which 16 were prospective and 15 were retrospective.

The indication for PPI varied between studies. PPI was defined as post-procedural or within 30 days after TAVI in most studies; however, some studies also included a minority of patients who experienced PPI after 30 days from the procedure.

The incidence of PPI ranged from 6.2% to 34.8% across the studies.

### All-cause death at long-term follow-up

**Figure 1:**
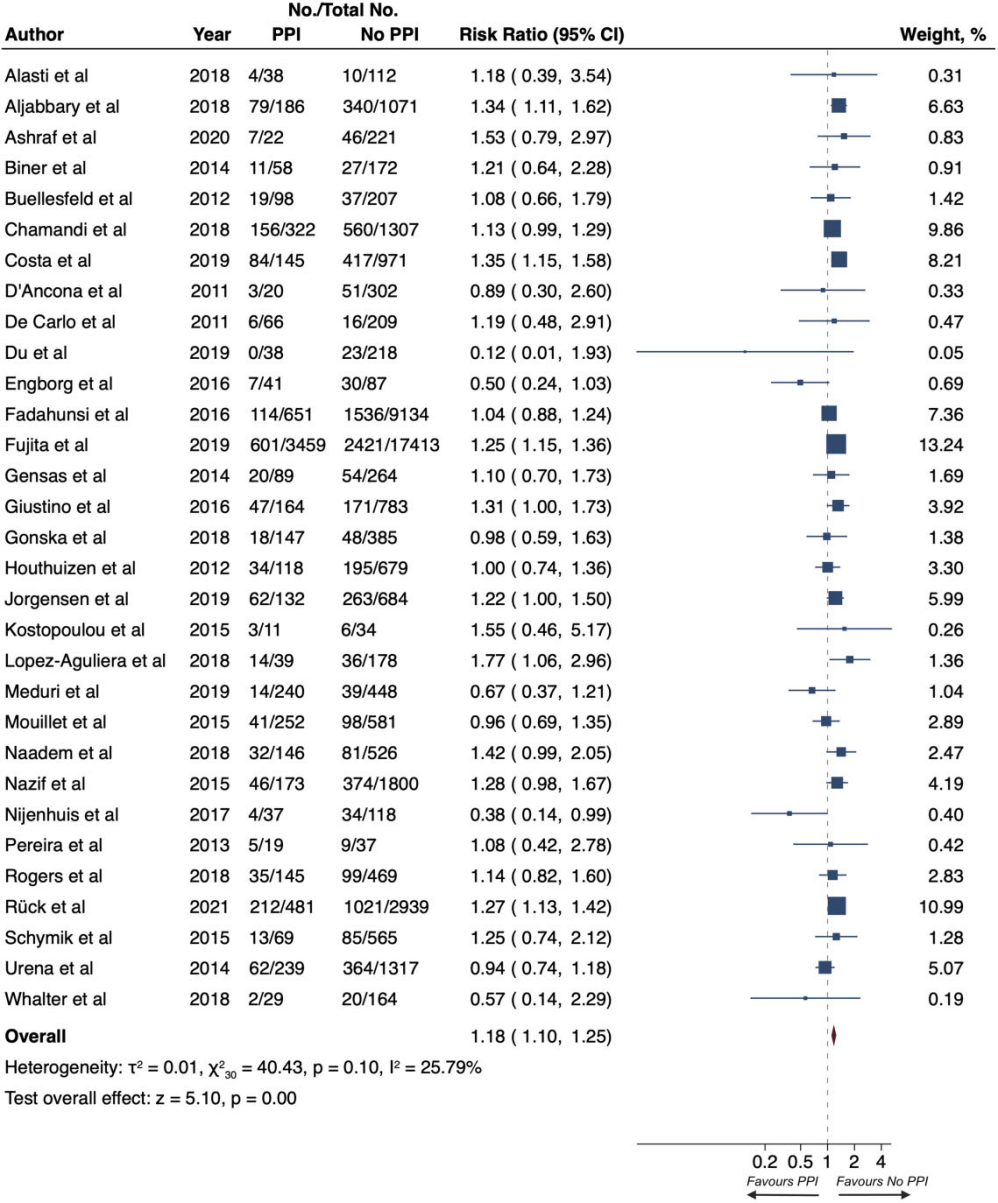
Risk of all-cause death at long-term follow-up. Squares represent risk ratios, with the size of the squares indicating weight of the studies and horizontal lines representing 95% CIs. The diamond represents the pooled risk ratio with the points of the diamond representing 95% CIs. CIs, confidence intervals.

Among 51 069 patients undergoing TAVI, the risk of all-cause death at LTF was higher for patients who experienced PPI (22.9% vs. 19.6%; RR 1.18, 95% CI 1.10–1.25, *P* < 0.001; *Figure 1*). The heterogeneity between the studies was moderate (*I*^2^ = 25.79%) and there was a potential publication bias detected by the Egger regression and funnel plot inspection (*P* = 0.015; Supplementary material online, *Figure S5*).

In the subgroup analysis performed in the 20 studies reporting the mortality risk of patients predicted by the STS-PROM score, the association between PPI and all-cause death risk at LTF was significant only in studies enrolling patients with high STS-PROM score (26.7% vs. 24.6%; RR, 1.25; 95% CI, 1.12–1.40; Supplementary material online, *Figure S1*), but not in those enrolling patients at intermediate (19.9% vs. 17.3%; RR, 1.11; 95% CI, 0.98–1.25; Supplementary material online, *Figure S1*) or low risk (47.0% vs. 38.5%; RR, 1.22; 95% CI, 1.00–1.50; Supplementary material online, *Figure S1*). It is worth noting, however, that the tendency of the results was similar in all three groups without a significant heterogeneity (*P* = 0.33; Supplementary material online, *Figure S1*).

### All-cause death at 1 year and 30 days

In a pooled analysis of 45 270 patients, those with PPI post-TAVI experienced an increased risk of all-cause death at 1 year (16.6% vs. 15.1%; RR 1.13, 95% CI 1.05–1.22; *P* < 0.001; Supplementary material online, *Figure S2*). There was low heterogeneity between studies (*I*^2^ = 8.37%) and a potential publication bias (*P* = 0.015; Supplementary material online, *Figure S5*). Conversely, the risk of all-cause death at 30 days, pooled from 40 806 patients, was not different between patients with PPI and without it (3.7% vs. 3.9%; RR 1.03, 95% CI 0.90–1.19; *P* = 0.66; Supplementary material online, *Figure S3*). The heterogeneity across the studies was low (*I*^2^ = 0.00%) and no potential publication bias was detected (*P* = 0.334; Supplementary material online, *Figure S5*).

### Rehospitalization for heart failure at long-term follow-up and 1 year

**Figure 2:**
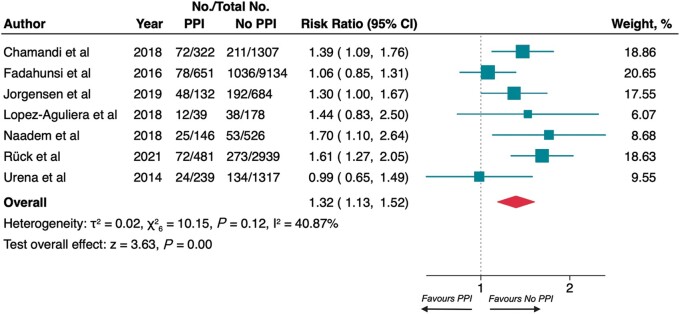
Risk of rehospitalization for heart failure at long-term follow-up. Squares represent risk ratios, with the size of the squares indicating weight of the studies and horizontal lines representing 95% CIs. The diamond represents the pooled risk ratio with the points of the diamond representing 95% CIs. CIs, confidence intervals.

The pooled results among 18 095 patients demonstrated that PPI was associated with rehospitalization for heart failure at LTF (16.5% vs. 12.0%; RR 1.32, 95% CI 1.13–1.52; *P* < 0.001; *Figure 2*). The heterogeneity across the studies was moderate (*I*^2^ = 40.87%) and no significant asymmetry was detected (*P* = 0.752; Supplementary material online, *Figure S5*).

The same results were detected at 1 year (12.2% vs. 10.7%; RR 1.26, 95% CI 1.02–1.56; *P* = 0.03; Supplementary material online, *Figure S4*) among 14 867 patients; the heterogeneity was moderate (*I*^2^ = 42.48%), no potential publication bias was disclosed (*P* = 0.766; Supplementary material online, *Figure S5*).

### Stroke at 1 year and myocardial infarction at 1 year

All studies reporting data regarding stroke and myocardial infarction in the LTF had 1 year observation time so that both endpoint evaluations at LTF and 1 year were coincident.

**Figure 3:**
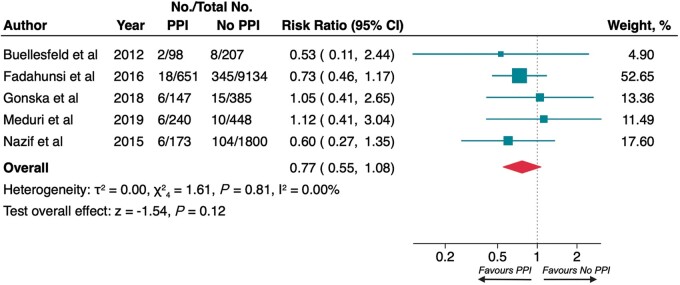
Risk of stroke at 1 year. Squares represent risk ratios, with the size of the squares indicating weight of the studies and horizontal lines representing 95% CIs. The diamond represents the pooled risk ratio with the points of the diamond representing 95% CIs. CIs, confidence intervals.

**Figure 4:**
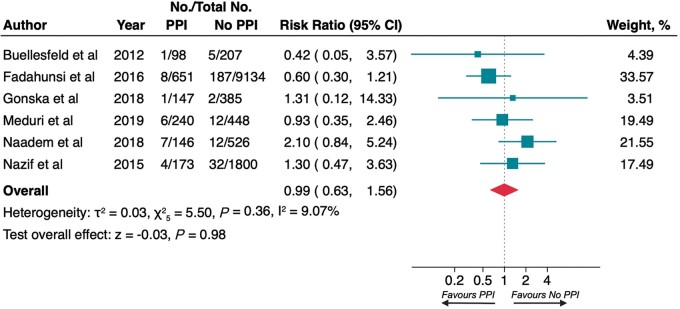
Risk of myocardial infarction at 1 year. Squares represent risk ratios, with the size of the squares indicating weight of the studies and horizontal lines representing 95% CIs. The diamond represents the pooled risk ratio with the points of the diamond representing 95% CIs. CIs, confidence intervals.

At 1 year, no difference in risk of stroke (2.9% vs. 4.0%; RR 0.77, 95% CI 0.55–1.08; *P* = 0.12; *Figure 3*) and myocardial infarction (1.9% vs. 2.0%; RR 0.99, 95% CI 0.63–1.56; *P* = 0.98; *Figure 4*) was observed between patients who required PPI and controls. The heterogeneity between studies was low (*I*^2^ 0.00% and 9.07%, respectively) and there was not significant publication bias (*P* = 0.383, *P* = 0.980; Supplementary material online, *Figure S5*).

### Sensitivity and meta-regression analyses

Sensitivity analyses performed comparing primary and secondary analyses obtained similar results (Supplementary material online, *Table S5*).

Meta-regression analyses showed no significant relation between most covariates and long-term outcomes. However, NOS was inversely associated with a higher risk of rehospitalization for heart failure at LTF related to PPI.

## Discussion

The need for post-procedural PPI represents the Achille’s heel of TAVI. In this meta-analysis of 31 observational studies, we found that patients who underwent PPI post-TAVI had a greater risk of all-cause death and rehospitalization for heart failure at 1 year and long-term follow-up.

Previous meta-analyses were contradictory about the relationship between PPI post-TAVI and the risk of worse clinical outcomes: indeed, some showed a significant impact in hard clinical endpoints such as all-cause death^14,^^18^ and rehospitalization for heart failure,^14^ while most did not show a significant clinical worsening;^15–17^ of note, the follow-up period was mostly limited to 1 year.

To the best of our knowledge, this is the most updated meta-analysis, with the largest sample size that evaluates clinical outcomes at various follow-up times (including long-term follow-up); moreover, this is the first article performing a subgroup analysis according to the preoperative procedural risk.

The ventricular dyssynchrony related to the right ventricular pacing could play an important role in increasing the risk of all-cause death among patients with PPI.^50–52^ Furthermore, it might also explain the increased risk of rehospitalization for heart failure and the absence of an impact of PPI on short-term mortality (at 30 days). In this regard, Nadeem *et al*.^42^ documented that patients with right ventricular pacing >40% had a higher risk of heart failure compared with those who experienced a lower right ventricular pacing burden.

Unfortunately, the few data and the variable pacing percentage cut-offs adopted in the various studies did not allow to perform a pooled analysis to evaluate the influence of the aforementioned variable on clinical outcomes. By this logic, different types of ventricular pacing (such as cardiac resynchronization therapy or His pacing) or proper device programming, could have a beneficial impact on the prognosis of patients undergoing post-procedural PPI.

Furthermore, higher mortality observed in patients who underwent PPI after TAVI may be related to different causes, both cardiac and non-cardiac, so that PPI could represent only a simple bystander. For instance, worse outcomes related to PPI could also be explained by the mechanical or ischaemic injury to the conduction system that can occur during TAVI. Indeed, new-onset persistent left bundle branch block was found to be associated with an increased risk of all-cause death and rehospitalization for heart failure.^14^

Interestingly, the significantly higher risk of all-cause death at LTF associated with PPI was confined to studies enrolling patients at high preoperative risk of mortality (≥8%, according to the STS-PROM score), while it was of borderline significance in those enrolling patients at medium or low risk. These findings are probably affected by the greater multimorbidity burden of high-risk patients undergoing TAVI and, therefore, by the relatively short follow-up available; indeed, among patients with a lower multimorbidity burden, a longer follow-up would be needed to establish the association between PPI and long-term mortality. On the other hand, the benefit of TAVI over SAVR regarding procedural risks is probably smaller in the group with STS-PROM score < 8% and so, in these patients, the disadvantage related to the long-term impact of PPI may be larger. Probably, with the extension of the indications for TAVI also in low-risk patients, the recruitment of patients with a lower average age will help to highlight this issue. However, it should be emphasized that the STS-PROM score may not intercept all comorbidities that could impact long-term mortality and this may limit the interpretation of these results.

Although right ventricular pacing is associated with an increased risk of atrial fibrillation,^53^ in our study PPI was not associated with a higher risk of stroke at 1-year follow-up. This may be caused by the relatively short follow-up of the studies included and the presence of confounding factors (such as post-procedural atrial fibrillation and antithrombotic therapy) that were not adjusted during the analysis.

Recently, several studies have documented the presence of electrical, anatomical, and procedural predictors of PPI after TAVI such as age, pre-existing conduction abnormalities, calcification of the left ventricular outflow tract, the use of self-expanding valve type, balloon valvuloplasty, and valve implantation depth.^54^ Other factors were found to predict a high percentage of long-term pacing in patients who experienced post-TAVI PPI such as high left ventricular outflow tract diameter ratio, high aortic annulus diameter ratio, new onset of left bundle branch block, time to PPI >2 days, and therapy with beta-blockers.^55^ Consequently, the choice of intervention modality in patients with AS should take into account the factors mentioned above.

In light of the results of this meta-analysis, strategies aimed to reduce the incidence of PPI might have an impact on the long-term outcomes of patients undergoing TAVI. Recently, higher valve implantation showed a reduction in conduction abnormalities and permanent pacemaker requirement, without compromising procedural safety or valve haemodynamic.^56^ In addition, other specific changes to the TAVI implementation techniques have been proposed.^57,^^58^ Findings from other ongoing trials are needed to strengthen this evidence.

### Limitations

Our meta-analysis has some limitations. Since systematic reviews and meta-analyses rely on the quality of included studies, we could only use observational studies, many of them with retrospective follow-up. Besides, the lack of pacing frequency data did not allow us to judge the influence of this variable on outcomes. Also, the lack of single patient-level data regarding the mortality outcome has foreclosed subgroup analyses and the possibility of establishing whether the need for PPI is an independent predictor of worse outcomes. Further, indications for PPI were different in the various studies limiting results reproducibility. Finally, over the period time of the present meta-analysis, there have been some important changes and evolution in the design and technique of TAVI procedure.

## Conclusion

Patients who underwent PPI after TAVI had an increased risk of all-cause death and rehospitalization for heart failure 1 year after the implantation and at the long-term follow-up. On the other hand, PPI did not modify the risk of all-cause death after 30 days, stroke, and myocardial infarction.

## Supplementary material

Supplementary material is available at *Europace* online.

## Supplementary Material

euac008_Supplementary_Data
