# Supplementary material for: Long-term clinical impact of permanent pacemaker implantation in patients undergoing transcatheter aortic valve implantation: a systematic review and meta-analysis
Source: Europace. 2022 Feb 9;24(7):1127–36. doi: 10.1093/europace/euac008 (PMC9460982; doi:10.1093/europace/euac008)
Supplement: euac008_Supplementary_Data [file euac008_supplementary_data.docx]

**Supplementary Materials**

**Supplementary Table 1. Full electronic search strategy through October 15th, 2021.**

**Supplementary Table 2. MOOSE Flow-Chart.**

**Supplementary Table 3. Clinical and procedural characteristics of the population in the included studies.**

**Supplementary Table 4. Assessment of study quality using the Newcastle-Ottawa Scale.**

**Supplementary Table 5. Results of Sensitivity Analyses.**

**Supplementary Table 6. Meta-Regressions for Long-Term Outcomes: P-Values for Interaction.**

**Supplementary Figure 1. Risk of all-cause death at long-term follow-up in patients with PPI after TAVI according to the mortality risk of patients predicted by STS-PROM score.**

**Supplementary Figure 2. Risk of all-cause death at 1 year.**

**Supplementary Figure 3. Risk of all-cause death at 30 days.**

**Supplementary Figure 4. Risk of rehospitalization for heart failure at 1 year.**

**Supplementary Figure 5. Funnel plots.**

**Supplementary Table 1. Full electronic search strategy through October 15th, 2021**

**PubMed**

(TAVI OR TAVR OR "Transcatheter Aortic Valve"[MeSH] OR Transcatheter Aortic Valve OR "Percutaneous Aortic Valve"[MeSH] OR Percutaneous Aortic Valve) AND ("Permanent Pacemaker Implantation"[MeSH] OR Permanent Pacemaker Implantation) AND ("Mortality"[MeSH] OR Mortality OR "Death"[MeSH] OR Death)

**EMBASE**

('tavi'/exp OR tavi OR tavr OR 'transcatheter aortic valve'/exp OR 'transcatheter aortic valve' OR (transcatheter AND aortic AND ('valve'/exp OR valve)) OR 'percutaneous aortic valve'/exp OR 'percutaneous aortic valve' OR (percutaneous AND aortic AND ('valve'/exp OR valve))) AND ('permanent pacemaker implantation' OR (permanent AND ('pacemaker'/exp OR pacemaker) AND ('implantation'/exp OR implantation))) AND ('mortality'/exp OR mortality OR 'death'/exp OR death)

**Supplementary Table 2. MOOSE Flow-Chart**

Records identified through

PubMed searching
(n = **616**)

Records identified through

EMBASE sources
(n = **1450**)

## Identification

## Included

Records excluded

(n = 2003)

Records screened
(n = **2066**)

## Screening

Full-text excluded (n = 32)

14 Not fit incl./excl. criteria

2 Duplicated cohorts

16 No Full-text available

Full-text screened
(n = **63**)

## Eligibility

Studies included in quantitative synthesis (meta-analysis)
(n = **31**)

**Supplementary Table 3. Clinical and procedural characteristics of the population in the included studies.**

| **Author** | **Year** | **Age (years)** | **Male (%)** | **Atrial Fibrillation (%)** | **Diabetes Mellitus (%)** | **Coronary artery disease (%)** | **LVEF** | **Type of PPI (%)** | **Indications**  **for PPI** | **STS-PROM (%)** |
| --- | --- | --- | --- | --- | --- | --- | --- | --- | --- | --- |
| Alasti et al | 2018 | 83.6 ± 5.6 | 69 (45.4) | 41 (27.3) | 28 (18.7) | NA | 52.2 ± 17.7 | Dual-chamber (97.4), Single-chamber (2.6) | Advanced AVB, AF with slow ventricular  rate, ventricular standstill, first-degree AVB with LBBB, non-TAVI related indication (such as sinus node dysfunction) | NA |
| Aljabbary et al | 2018 | 82.3 ± 7.2 | 666 (52.7) | NA | 593 (47.0) | 910 (72.1) | 186 (14.7) | Single- or dual-chamber (96.3),  ICD (1.6),  CRT-D (2.1) | NA | 8.6 ± 7.6 |
| Ashraf et al | 2020 | 82.0 ± 7.4 | 140 (57.6) | 75 (30.9) | 59 (24.3) | 104 (42.8) | 57.3 ± 12.8 | Dual-chamber (100) | 2018 ACC/AHA/HRS guidelines | NA |
| Biner et al | 2014 | 83.0 ± 5.0 | 88 (38.3) | 43 (18.7) | 75 (32.9) | NA | 58 ± 10 | NA | Pre-TAVI RBBB, post-TAVI advanced AVB, alternating BBB, new LBBB with PR-interval prolongation ≥ 280 ms | NA |
| Buellesfeld et al | 2012 | 82.6 ± 6.1 | 126 (41.3) | 67 (22.0) | 77 (25.3) | 166 (54.4) | 51.3 ± 15.0 | NA | Advanced AVB, new-onset LBBB with dynamic PR interval prolongation > 300 ms, AF with inadequate ventricular escape rhythm | NA |
| Chamandi et al | 2018 | 81.2 ± 6.8 | 949 (58.3) | 347 (21.3) | 561 (34.4) | 691 (42.4) | 56.2 ± 12.8 | Dual-chamber (56.8),  Single-chamber (40.7),  Unspecified (2.5) | 2012 ACC/AHA/HRS guidelines, LBBB with PR interval prolongation (>200 ms) at the discretion of the physician | 7.0 ± 5.4 |
| Costa et al | 2019 | 80.9 ± 5.3 | 468 (41.9) | 177 (15.9) | 323 (28.9) | NA | 53.3 ± 11.0 | NA | 2013 ESC guidelines | 4.4 ± 3.4 |
| D’Ancona et al | 2011 | 79.1 ± 8.4 | 107 (33.2) | 93 (28.9) | 80 (2.5) | 192 (59.6) | 50.5 ± 14.9 | NA | Post-TAVI complete AVB, symptomatic bradycardia (on the fifth postoperative day if with ventricular replacement rhythm or on the third postoperative day if without ventricular replacement rhythm) | 18.5 ± 16.0 |
| De Carlo et al | 2011 | 82.4 ± 5.9 | 128 (46.5) | NA | NA | NA | 51.8 ± 11.8 | NA | 2007 ESC guidelines | NA |
| Du et al | 2019 | 76.5 ± 6.1 | 148 (57.8) | NA | 55 (21.8) | NA | 53.1 ± 13.8 | NA | 2012 ACC/AHA/HRS guidelines | 7.1 ± 5.9 |
| Engborg et al | 2016 | 80.7 ± 5.5 | 57 (44.5) | 32 (25.0) | 23 (18.0) | NA | 50.3 ± 13.4 | NA | Advanced AVB, sick sinus syndrome new LBBB with first degree AVB | NA |
| Fadahunsi et al | 2016 | 84 (78-88) | 4621 (47.2) | 3627 (37.1) | 3407 (34.8) | 2670 (27.3) | 57.9 (49-65) | NA | NA | 6.7 (4.5 - 11.2) |
| Fujita et al | 2019 | 81 (78 - 85) | 9442 (45.2) | 5829 (27.9) | 6840 (32.8) | 11205 (53.7) | NA | NA | NA | 4.4 (3.1–6.4) |
| Gensas et al | 2014 | 81.5 ± 7.6 | 164 (46.5) | NA | 113 (32.0) | NA | NA | NA | NA | 14.4 ± 10.2 |
| Giustino et al | 2016 | 81.0 ± 7.0 | 485 (51.2) | 206 (21.2) | 947 (28.5) | NA | 52.2 ± 13.8 | NA | 2012 ACC/AHA/HRS guidelines, LBBB with PR interval prolongation (>200 ms) at the discretion of the physician | 8.6 ± 6.6 |
| Gonska et al | 2018 | 80.4 ± 5.9 | 288 (47.1) | 220 (35.9) | 183 (29.9) | 374 (61.1) | 57.3 ± 15.2 | Dual-chamber (73.2),  Single-chamber (20.2),  ICD (1.2),  CRT-P (3),  CRT-D (2.3) | NA | 6.5 ± 4.9 |
| Houthuizen et al | 2012 | NA | NA | NA | NA | NA | NA | NA | NA | NA |
| Jorgensen et al | 2018 | 81 (75 -85) | 209 (25.6) | 280 (34.3) | 169 (20.7) | 399 (48.9) | NA | Single-dual chamber (92.4),  ICD (2.3),  CRT (6.8) | NA | 3.2 (2.2 - 4.9) |
| Kostopoulou et al | 2015 | 81.0 ± 5.0 | 27 (60.0) | NA | 12 (26.7) | 20 (44.4) | 49 ± 11 | Dual-chamber (90),  Single-chamber (10) | Advanced AVB, new LBBB with  infrahisian conduction delay (defined as HV > 70 ms) | NA |
| Lopez-Aguliera et al | 2018 | 78.0 ± 6.5 | 102 (47.0) | 54 (24.9) | 64 (29.5) | NA | 58.7 ± 12.1 | Dual-chamber (30.8),  Single-chamber (69.2) | Advanced AVB, LBBB or new first-degree AVB with persistent severe bradycardia (<40 bpm) or syncope | 11.4 ± 10.1 |
| Meduri et al | 2019 | 82.3 ± 7.7 | 345 (49.0) | 202 (28.7) | 218 (31.0) | 497 (70.6) | NA | NA | NA | 6.6 ± 4.0 |
| Mouillet et al | 2015 | 82.1 ± 7.3 | 489 (58.7) | NA | 215 (24.9) | 403 (46.6) | 52.9 ± 14.1 | NA | NA | 14.1 ± 11.2 |
| Naadem et al | 2018 | 81.3 (43-102) | 337 (50.1) | 205 (30.5) | 276 (41.1) | NA | 53.2 (10-80) | NA | NA | 7.6 (0.74-34) |
| Nazif et al | 2015 | 84.3 ± 7.2 | 955 (48.4) | NA | 715 (36.2) | 1504 (76.2) | 53.9 | Dual-chamber (75.7),  Single-chamber (19.7),  ICD (0.6)  CRT-P (2.9)  CRT-D (0.6),  Unkown (0.6) | Advanced AVB, symptomatic bradycardia, sick sinus syndrome | 11.3 ± 4.0 |
| Nijenhuis et al | 2017 | 80.0 ± 7.0 | 78 (50.3) | 94 (60.7) | 38 (24.5) | 89 (57.4) | 59 ± 14.5 | Dual-chamber (65)  Single-chamber (30)  CRT-D (5) | 2013 ESC guidelines | 5.5 ± 3.5 |
| Pereira et al | 2013 | 79.3 ± 6.5 | 30 (46.2) | 26 (40.0) | 25 (38.5) | 29 (44.6) | NA | Dual-chamber (68.4)  Single-chamber (31.5) | 2007 ESC guidelines | NA |
| Rogers et al | 2018 | 83.0 ± 9.0 | 283 (46.1) | 221 (36.0) | 203 (33.1) | NA | 54 ± 13 | NA | NA | 8.6 ± 4.6 |
| Rück et al | 2021 | 81.3 ± 7.2 | 1698 (49.6) | 1375 (40.2) | 988 (28.9) | NA | NA | NA | NA | NA |
| Schymik et al | 2014 | 82.0 ± 4.5 | 243 (38.3) | NA | 214 (33.8) | 357 (56.3) | 59.3 ± 13.1 | NA | 2013 ESC guidelines | NA |
| Urena et al | 2014 | 80.2 ± 7.5 | 740 (47.6) | 434 (27.9) | 485 (31.2) | 877 (56.4) | 55.2 ± 13.9 | Dual-chamber (59.8)  Single-chamber (40.2) | 2008 ACC/AHA/HRS guidelines, LBBB with PR interval prolongation (>200 ms) at the discretion of the physician | 7.6 ± 5.3 |
| Walther et al | 2018 | 83.1 ± 4.7 | 45 (22.7) | 44 (22.2) | 58 (29.3) | 118 (59.6) | NA | NA* | NA | 5.8 ± 3.4 |

Abbreviations: ACC/AHA/HRS, American College of Cardiology/American Heart Association/Heart Rhythm Society; AF, Atrial Fibrillation; AVB, Atrioventricular Block; CRT-D, Cardiac Resynchronization Therapy – Defibrillator; CRT-P, Cardiac Resynchronization Therapy – Pacemaker; ESC, European Society of Cardiology; ICD, Implantable Cardiac Defibrillator; LBBB, Left Bundle Brunch Block; LVEF, Left Ventricular Ejection Fraction; NA, Not available; PPI, Permanent pacemaker implantation; RBBB, Right Bundle Brunch Block; STS-PROM, Society of Thoracic Surgeon – Predicted Risk of Mortality; TAVI, Transcatheter Aortic Valve Implantation.

*Analysis was performed on 29 patients that experienced PPI during hospitalization; 33 patients experienced PPI within 1 year after TAVI: dual-chamber (57.6), single-chamber (24.2), CRT (6.1), unknown (12.1).

**Supplementary Table 4. Assessment of study quality using the Newcastle-Ottawa Scale**

| **Author** | **Selection** | | | | **Comparability of cohorts** | **Outcome** | | | **Total score** |
| --- | --- | --- | --- | --- | --- | --- | --- | --- | --- |
|  | **Exposed cohort representativeness** | **Non-exposed cohort selection** | **Exposure ascertain-ment** | **Absence of outcome at baseline** |  | **Outcome ascertainment** | **Long enough follow-up** | **Follow-up adequacy** |  |
| Alasti et al | 1 | 1 | 1 | 1 | 0 | 1 | 1 | 0 | **6** |
| Aljabbary et al | 1 | 1 | 1 | 1 | 0 | 1 | 1 | 0 | **6** |
| Ashraf et al | 1 | 1 | 1 | 1 | 1 | 1 | 1 | 1 | **8** |
| Biner et al | 1 | 1 | 1 | 1 | 0 | 1 | 1 | 0 | **6** |
| Buellesfeld et al | 1 | 1 | 1 | 1 | 2 | 1 | 1 | 1 | **9** |
| Chamandi et al | 1 | 1 | 1 | 1 | 1 | 1 | 1 | 0 | **7** |
| Costa et al | 1 | 1 | 1 | 1 | 2 | 1 | 1 | 0 | **8** |
| D’Ancona et al | 1 | 1 | 1 | 1 | 2 | 1 | 1 | 1 | **9** |
| De Carlo et al | 1 | 1 | 1 | 1 | 0 | 1 | 1 | 0 | **6** |
| Du et al | 1 | 1 | 1 | 1 | 0 | 1 | 1 | 0 | **6** |
| Engborg et al | 1 | 1 | 1 | 1 | 2 | 1 | 1 | 1 | **9** |
| Fadahunsi et al | 1 | 1 | 1 | 1 | 2 | 1 | 1 | 1 | **9** |
| Fujita et al | 1 | 1 | 1 | 1 | 2 | 1 | 1 | 1 | **9** |
| Gensas et al | 1 | 1 | 1 | 1 | 2 | 1 | 1 | 0 | **8** |
| Giustino et al | 1 | 1 | 1 | 1 | 2 | 1 | 1 | 0 | **8** |
| Gonska et al | 1 | 1 | 1 | 1 | 2 | 1 | 1 | 0 | **8** |
| Houthuizen et al | 1 | 1 | 1 | 1 | 0 | 1 | 1 | 0 | **6** |
| Jorgensen et al | 1 | 1 | 1 | 1 | 2 | 1 | 1 | 0 | **8** |
| Kostopoulou et al | 1 | 1 | 1 | 1 | 0 | 1 | 1 | 0 | **6** |
| Lopez-Aguliera et al | 1 | 1 | 1 | 1 | 1 | 1 | 1 | 0 | **7** |
| Meduri et al | 1 | 1 | 1 | 1 | 1 | 1 | 1 | 0 | **7** |
| Mouillet et al | 1 | 1 | 1 | 1 | 2 | 1 | 1 | 0 | **8** |
| Naadem et al | 1 | 1 | 1 | 1 | 2 | 1 | 1 | 0 | **8** |
| Nazif et al | 1 | 1 | 1 | 1 | 2 | 1 | 1 | 0 | **8** |
| Nijenhuis et al | 1 | 1 | 1 | 1 | 2 | 1 | 1 | 0 | **8** |
| Pereira et al | 1 | 1 | 1 | 1 | 0 | 1 | 1 | 0 | **6** |
| Rogers et al | 1 | 1 | 1 | 1 | 0 | 1 | 1 | 0 | **6** |
| Rück et al | 1 | 1 | 1 | 1 | 1 | 1 | 1 | 1 | **8** |
| Schymik et al | 1 | 1 | 1 | 1 | 2 | 1 | 1 | 0 | **8** |
| Urena et al | 1 | 1 | 1 | 1 | 2 | 1 | 1 | 1 | **9** |
| Walther et al | 1 | 1 | 1 | 1 | 0 | 1 | 1 | 0 | **6** |

**Supplementary Table 5. Results of Sensitivity Analyses**

| **Endpoint** | **Model** | **Risk Ratio (95% CI)** |
| --- | --- | --- |
| All-cause death at long-term follow-up | Random-effects RR | 1.18 (1.10-1.25) |
|  | Random-effect OR | 1.23 (1.11-1.35) |
|  | Fixed-effects RR | 1.18 (1.13-1.23) |
| All-cause death at 1 year | Random-effects RR | 1.13 (1.05-1.22) |
|  | Random-effect OR | 1.15 (1.04-1.26) |
|  | Fixed-effects RR | 1.15 (1.08-1.22) |
| All-cause death at 30 days | Random-effects RR | 1.03 (0.90-1.19) |
|  | Random-effect OR | 1.03 (0.89-1.20) |
|  | Fixed-effects RR | 1.02 (0.89-1.18) |
| Rehospitalization for heart failure at long-term follow-up | Random-effects RR | 1.32 (1.13-1.52) |
|  | Random-effect OR | 1.39 (1.15-1.68) |
|  | Fixed-effects RR | 1.29 (1.16-1.44) |
| Rehospitalization for heart failure at 1 year | Random-effects RR | 1.26 (1.02-1.56) |
|  | Random-effect OR | 1.30 (1.02-1.64) |
|  | Fixed-effects RR | 1.20 (1.04-1.39) |
| Stroke at 1 year | Random-effects RR | 0.77 (0.55-1.08) |
|  | Random-effect OR | 0.76 (0.53-1.08) |
|  | Fixed-effects RR | 0.75 (0.54-1.06) |
| Myocardial infarction at 1 year | Random-effects RR | 0.99 (0.63-1.56) |
|  | Random-effect OR | 0.99 (0.62-1.57) |
|  | Fixed-effects RR | 0.91 (0.60-1.37) |
| Abbreviations: OR, Odds Ratio; RR, Risk Ratio. |  |  |

**Supplementary Table 6. Meta-Regressions for Long-Term Outcomes**

| **Variable** | **All-cause death at LTF** | | **Rehospitalization for HF at LTF** | | **Stroke** | | **Myocardial Infarction** | |
| --- | --- | --- | --- | --- | --- | --- | --- | --- |
|  | *Coefficient* | *p-value* | *Coefficient* | *p-value* | *Coefficient* | *p-value* | *Coefficient* | *p-value* |
| Age | -0.010 | 0.709 | -0.051 | 0.280 | -0.125 | 0.374 | -0.251 | 0.143 |
| Male sex | <0.001 | 0.709 | <-0.001 | 0.170 | <-0.001 | 0.719 | <-0.001 | 0.094 |
| Atrial Fibrillation | <0.001 | 0.739 | <-0.001 | 0.189 | <-0.001 | 0.504 | <-0.001 | 0.189 |
| Diabetes Mellitus | <0.001 | 0.674 | <-0.001 | 0.118 | <-0.001 | 0.712 | <-0.001 | 0.113 |
| CAD | <0.001 | 0.221 | <-0.001 | 0.140 | <-0.001 | 0.586 | <0.001 | 0.478 |
| LVEF | -0.017 | 0.065 | -0.008 | 0.486 | 0.059 | 0.517 | -0.163 | 0.112 |
| No. of self-expanding valves | <0.001 | 0.369 | <-0.001 | 0.090 | <-0.001 | 0.773 | <-0.001 | 0.129 |
| No. of balloon-expanding valves | <0.001 | 0.768 | <-0.001 | 0.059 | <-0.001 | 0.820 | <-0.001 | 0.099 |
| NOS | -0.019 | 0.574 | **-0.159** | **0.047** | -0.180 | 0.472 | -0.367 | 0.208 |
| Follow-up | <0.001 | 0.616 | 0.002 | 0.674 | - | - | - | - |
| Abbreviations: CAD, Coronary Artery Disease; LTF, Long-term follow-up; LVEF, Left Ventricular Ejection Fraction; NOS, Newcastle-Ottawa Scale. | | | | | | | | |

**Supplementary Figure 1. Risk of all-cause death at long-term follow-up in patients with PPI after TAVI according to the mortality risk of patients predicted by STS-PROM score**

**
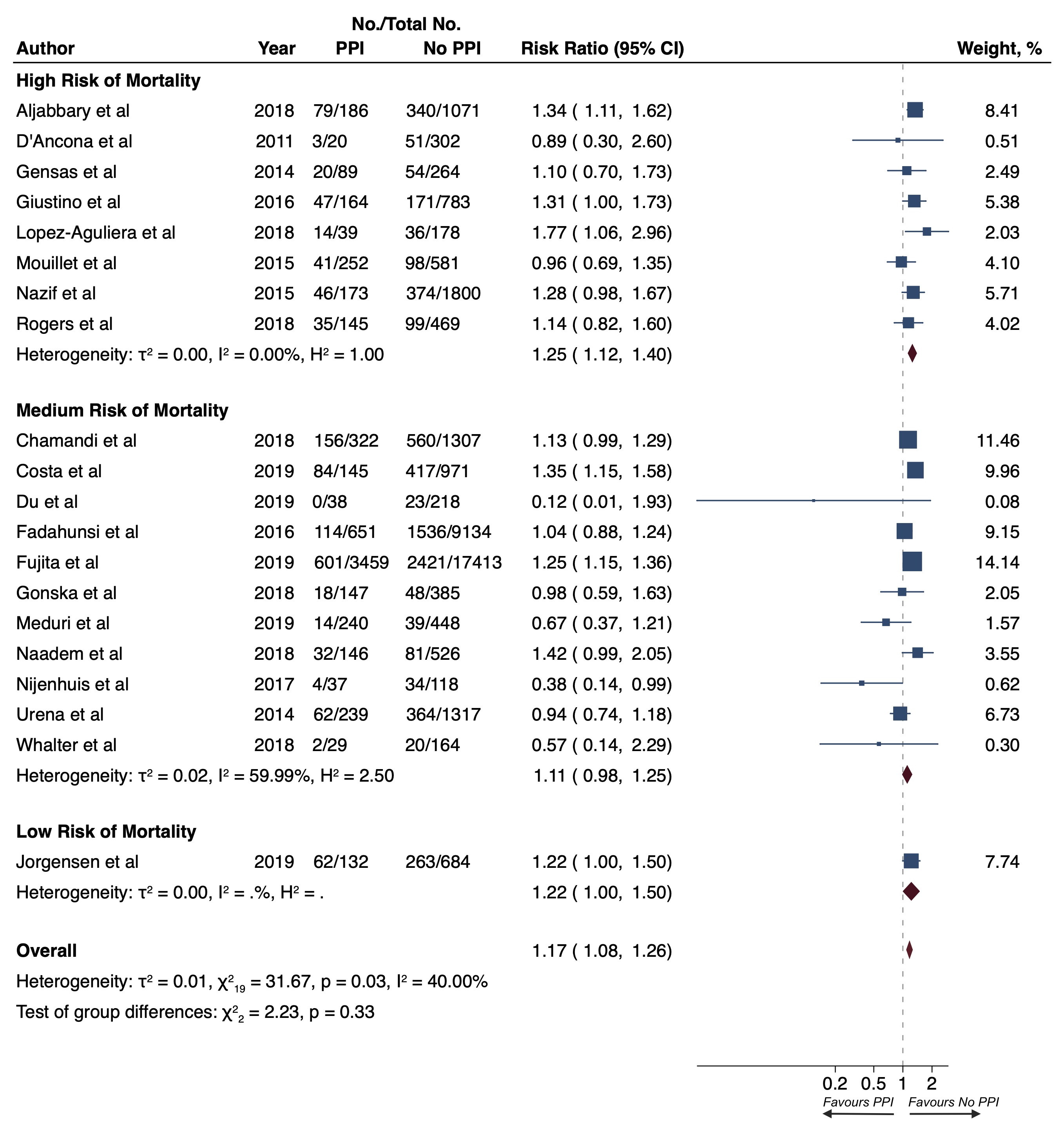
**

Legend: Squares represent risk ratios, with the size of the squares indicating weight of the studies and horizontal lines representing 95% CIs. The diamond represents the pooled risk ratio with the points of the diamond representing 95% CIs.

**Supplementary Figure 2. Risk of all-cause death at 1 year**

**
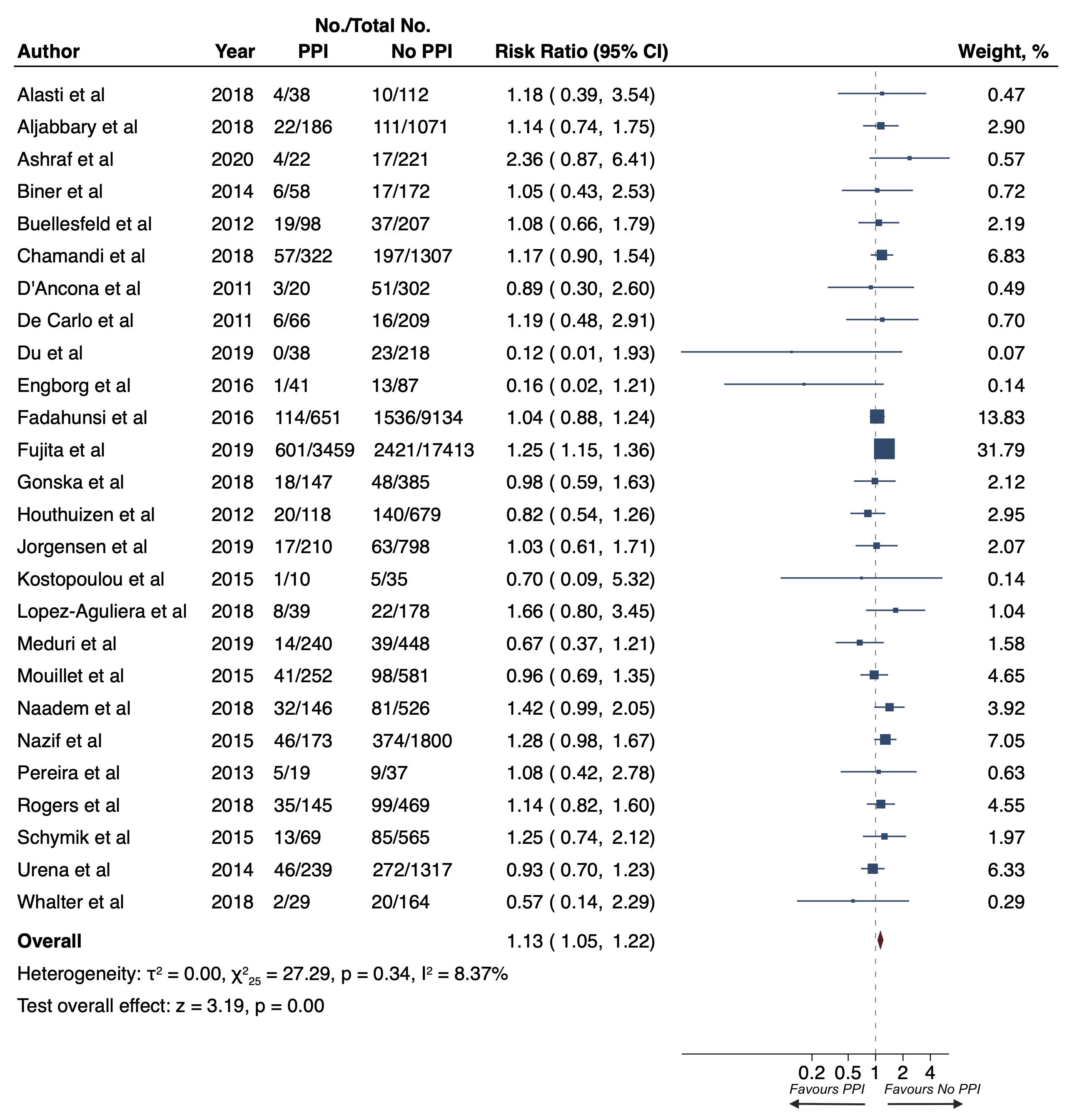
**

Legend: Squares represent risk ratios, with the size of the squares indicating weight of the studies and horizontal lines representing 95% CIs. The diamond represents the pooled risk ratio with the points of the diamond representing 95% CIs.

**Supplementary Figure 3. Risk of all-cause death at 30 days**

**
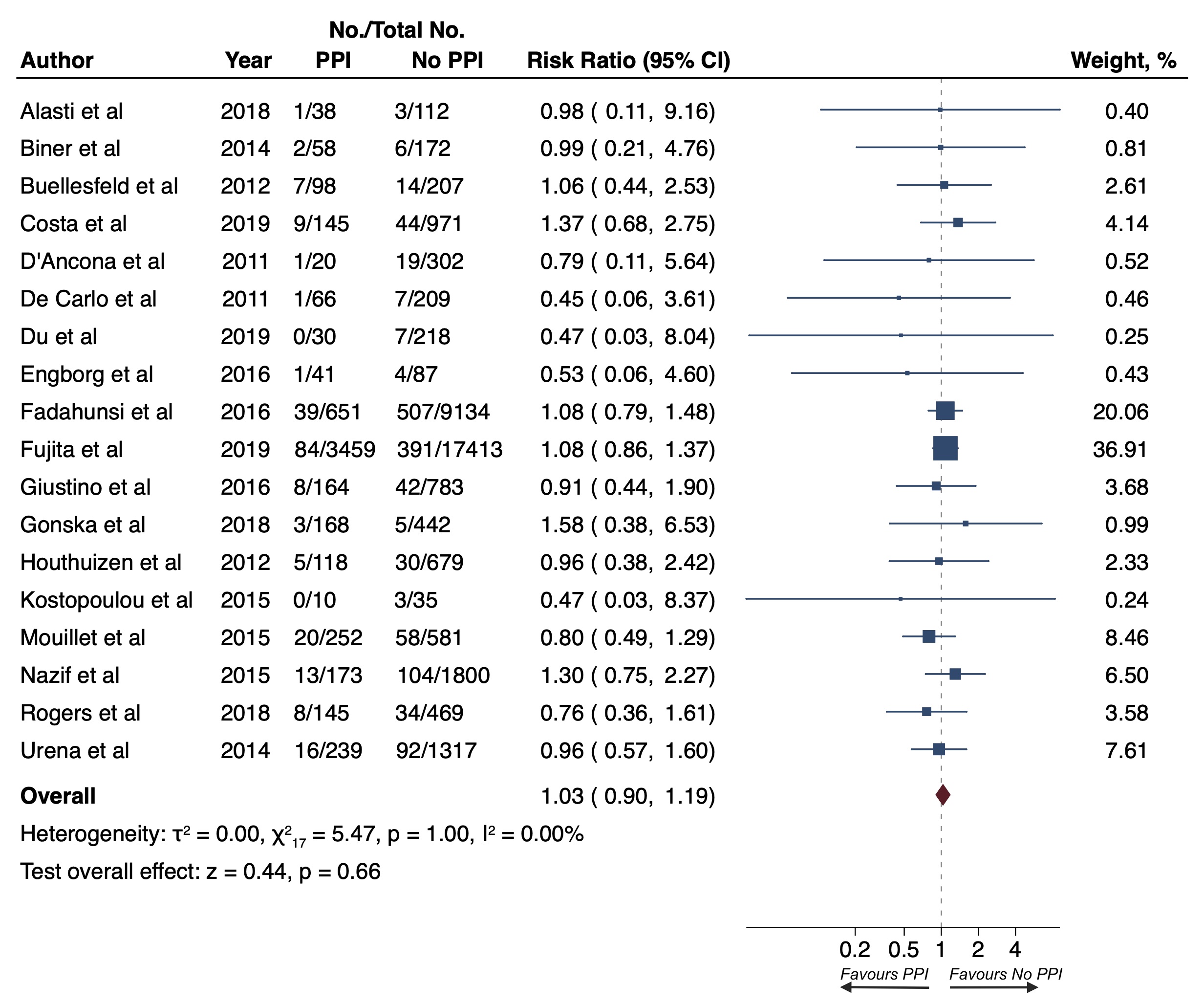
**

Legend: Squares represent risk ratios, with the size of the squares indicating weight of the studies and horizontal lines representing 95% CIs. The diamond represents the pooled risk ratio with the points of the diamond representing 95% CIs.

**Supplementary Figure 4. Risk of rehospitalization for heart failure at 1 year**

**
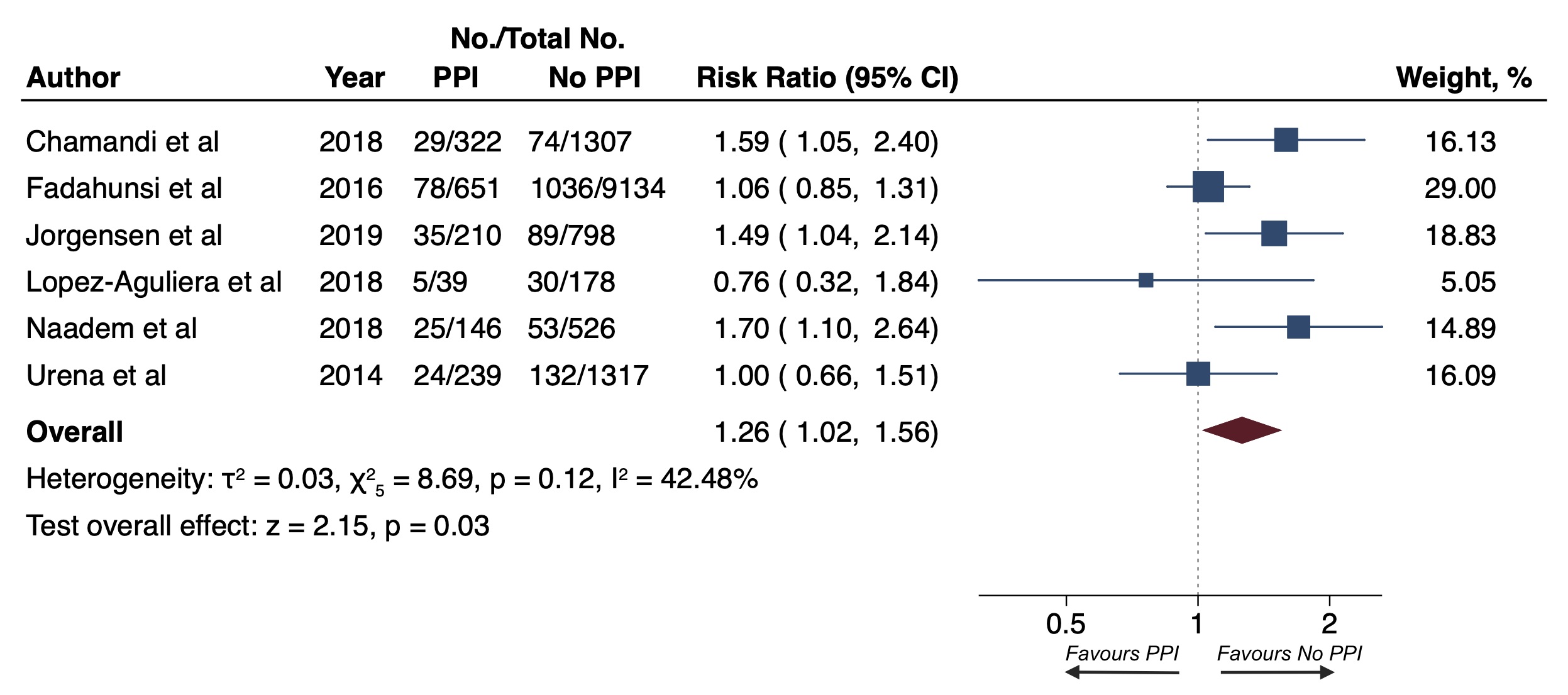
**

Legend: Squares represent risk ratios, with the size of the squares indicating weight of the studies and horizontal lines representing 95% CIs. The diamond represents the pooled risk ratio with the points of the diamond representing 95% CIs.

**Supplementary Figure 5. Funnel plots
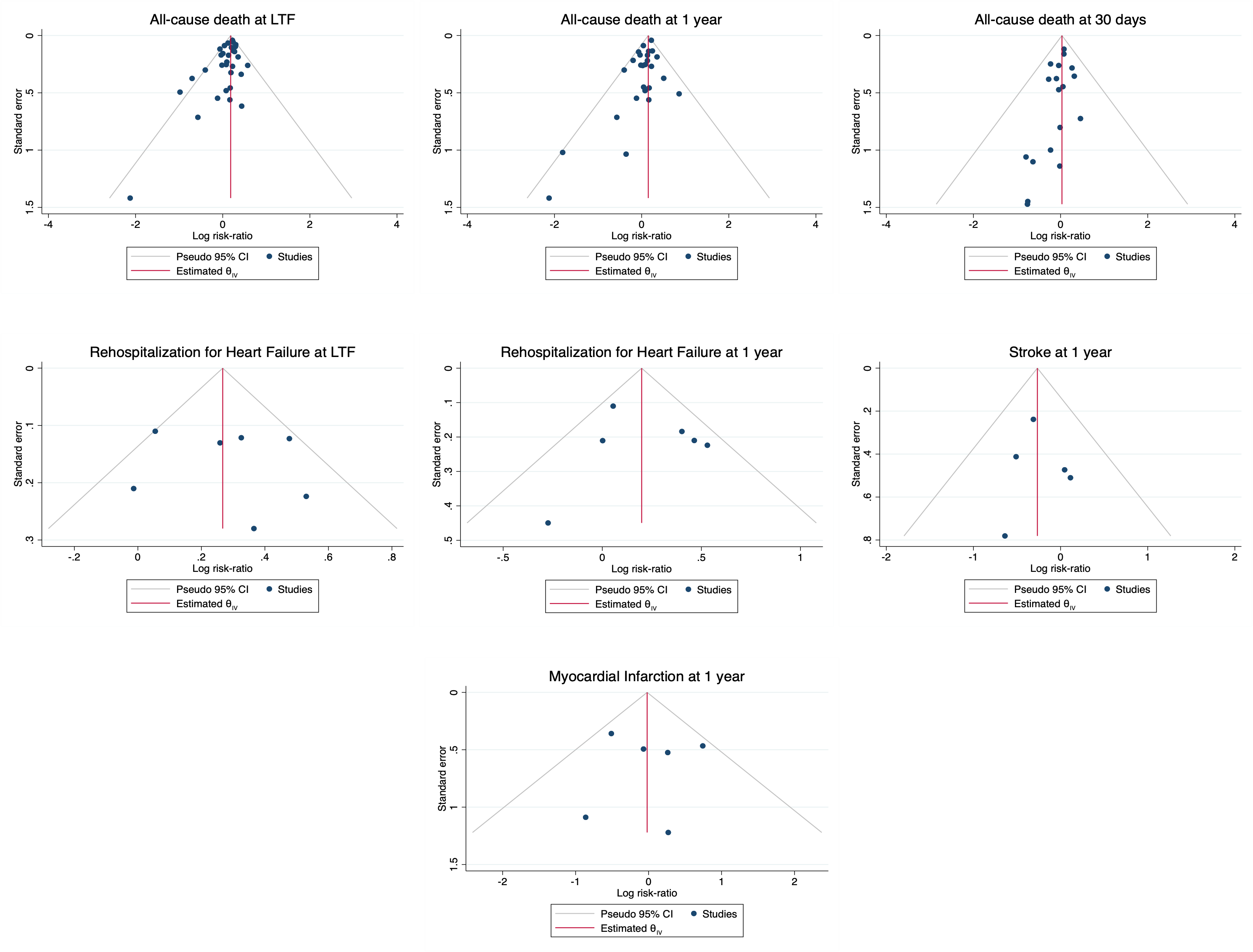
**
